# [Retracted] MicroRNA‑133a‑3p inhibits cell proliferation, migration and invasion in colorectal cancer by targeting AQP1

**DOI:** 10.3892/ol.2024.14812

**Published:** 2024-11-20

**Authors:** Bin Kong, Shipeng Zhao, Xianwu Kang, Bo Wang

Oncol Lett 22: 649, 2021; DOI: 10.3892/ol.2021.12910

Following the publication of this paper, it was drawn to the Editor's attention by a concerned reader that certain of the Transwell cell invasion and migration assay data shown in Figs. 2D and 5C respectively were strikingly similar to data that had already been published in different form in a pair of other articles written by different authors at a different research institutes.

Owing to the fact that the contentious data in the above article had already been published prior to its submission to *Oncology Letters*, the Editor has decided that this paper should be retracted from the Journal. The authors were asked for an explanation to account for these concerns, but the Editorial Office did not receive a reply. The Editor apologizes to the readership for any inconvenience caused.

